# “No thanks, I don’t want to see snakes again”: a qualitative study of pain management versus preservation of cognition in palliative care patients

**DOI:** 10.1186/s12904-020-00683-1

**Published:** 2020-11-29

**Authors:** Pete Wegier, Jaymie Varenbut, Mark Bernstein, Peter G. Lawlor, Sarina R. Isenberg

**Affiliations:** 1 Temmy Latner Centre for Palliative Care, Sinai Health, Toronto, Ontario Canada; 2 Lunenfeld-Tanenbaum Research Institute, Sinai Health, Toronto, Ontario Canada; 3 Department of Family and Community Medicine, University of Toronto, Toronto, Ontario Canada; 4 Division of Neurosurgery, Toronto Western Hospital, Toronto, Ontario Canada; 5 Bruyère Research Institute, Ottawa, Ontario Canada; 6 Division of Palliative Care, Department of Medicine, University of Ottawa, Ottawa, Ontario Canada; 7 Ottawa Hospital Research Institute, Ottawa, Ontario Canada; 8 Institute of Health Policy, Management, and Evaluation, Dalla Lana School of Public Health, University of Toronto, Toronto, Ontario Canada

**Keywords:** Pain management, Cognitive function, Palliative care, Decision making

## Abstract

**Background:**

Towards the end of life, use of opioid analgesics becomes more common in patients to control pain and improve quality of life. While pain medication may help manage pain, unwanted cognitive side effects are frequently noted. This balancing act presents a trade-off for patients between pain relief and adverse effects, where the desire to relieve pain must be evaluated against the desire to maintain cognitive clarity and may represent a difficult decision for patients receiving palliative care. Our goal was to understand how patients’ decision making about pain medications balances the pain relief from those medications against the cognitive decline often associated with them.

**Methods:**

We conducted qualitative semi-structured interviews with patients receiving home-based palliative care from a program in Toronto, Canada. Interview recordings were transcribed and analyzed using thematic analysis.

**Results:**

Thirty-one interviews were conducted. Some patients preferred cognitive preservation over pain management because of a sense that cognition is central to their identity, the desire to maintain lucidity, a desire to continue participating in work or hobbies, and fear of addiction. Conversely, some patients preferred pain management over cognitive preservation because of a desire to avoid suffering, an inability to sleep without medications, or an acceptance of some cognitive compromise. A few patients attempted to find balance through tapering drugs, limiting their use of breakthrough analgesic doses, or using alternative strategies.

**Conclusions:**

Decision making around pain and pain management is a highly preference-sensitive process—with no clear right or wrong decisions, only the preferences of each patient. The findings from this study may influence the design of future patient-facing decision aids around pain management. Future studies should pilot interventions to better assist patients with this decision.

**Supplementary Information:**

The online version contains supplementary material available at 10.1186/s12904-020-00683-1.

## Background

Pain management has emerged as a top priority within palliative populations [1–5]. Managing pain and symptoms is vital in chronic illnesses such as cancer and cardiovascular diseases, where 80 and 67% of patients, respectively, experience moderate to severe pain [6]. This high prevalence makes pain management a major focus of palliative and end-of-life care, where a curative approach is no longer the goal. The World Health Organization previously described the three-step analgesic ladder for pain relief—beginning with non-opioid analgesics, progressing to weak opioids, and on to strong opioids for moderate and severe pain [7]. Towards the end of life, the use of opioid analgesics becomes more common in patients to control pain and improve quality of life [8].

The high prevalence of pain and the focus on pain management has been correlated with large increases in the use of opioids to control these symptoms. In Canada in 2018, 12.3% of filled prescriptions at pharmacies were for an opioid [9]. While chronic pain medication may help manage pain, unwanted physical and cognitive side effects are frequently noted [5, 10, 11]. Some common adverse cognitive effects are mental clouding, sedation, and cognitive impairment [3, 11–14]. It has been observed that one third of cancer patients being treated with opioids likely experienced some level of cognitive dysfunction [15]. This balancing act presents a trade-off for patients between pain relief and adverse effects, where the desire to manage pain must be evaluated against the desire to preserve cognitive clarity.

The study of medical decision making is a growing field. The existing literature has shown many different approaches used—by both patients and healthcare providers—in medical choice situations [16–20]. Additionally, the overall process of decision making in pain management is complex, and input may be derived from multiple factors: the clinician’s assessment skills, prescribing knowledge, attitudes, communications skills and their relationship with the patient, including the degree to which shared decision-making occurs [21–23]; and the patient’s beliefs and attitudes, their capacity to self-manage medications and their socioeconomic characteristics such as education and racial identity [23–25]. Many patient concerns may impact decision making in cancer pain management and pose as attitudinal barriers to optimal opioid use: fear of addiction; fatalism—based on the association of opioids with end of life and fear of death; side-effects; opioid tolerance; concern that focussing on pain will distract from disease treatment or not allow monitoring of disease progression; desire to be a good patient; and concerns regarding compromised immunity [26, 27].

Decision making regarding opioids can be conceived as an approach-avoidance conflict. Past literature shows that decisions can be motivated by approach or avoidance arguments, where an individual can both be attracted to the potential benefits of an option (approach) while also being repelled by the potential drawbacks (avoidance) [28]. In our context, palliative care patients experiencing pain must make decisions about their level of opioid use to manage their pain. Patients would both be attracted to the pain relief which opioid use would provide, but also be repelled by the cognitive side effects which are common with opioids. The balancing of these options is at the centre of decision making in regard to pain management towards the end of one’s life. However, how patients feel about these options is not currently well understood. The current study was designed to examine palliative care patients’ concerns regarding the trade-offs between optimal pain control and cognitive preservation. We utilized qualitative interviews with patients in the home-based palliative care setting to understand the decision-making process around pain and pain management.

## Methods

### Study design

Qualitative semi-structured interviews were conducted with home-based palliative care patients through a home-based palliative care program in Toronto, Ontario—the Temmy Latner Centre for Palliative Care, a division of Sinai Health. Patients received palliative care in their homes. The study was approved by the research ethics board at Sinai Health in Toronto, Ontario (18–0021-E).

### Recruitment

We used a convenience sampling approach. Physicians from the Temmy Latner Centre for Palliative Care provided patient information for participants identified as being eligible for participation. Eligible patients were approached by a research staff face-to-face, informed about the study and consented to participate. Participants were eligible if they were: (1) 18 years of age or older, and (2) English speaking. Interviews were conducted at a time and location convenient for the study participants. Recruitment continued until it was believed that data saturation was reached through regular reviews of that transcripts by the interviewer (JV).

### Data collection

Interviews were conducted in English and were administered face-to-face. One interviewer (JV) completed the study interviews after obtaining consent, explaining the goals of the study, and recording patient demographic information. Questions such as “how would you describe the level of pain you are currently experiencing?”, “what side effects would you be willing to tolerate?”, and “what factors did you consider when making decisions about your pain management medications” were administered to the study participants. We developed the interview guide, which appears in Additional file 1. Interviews were conducted once with each participant, in the participants’ home and JV took field notes during the interviews. Interviews were audio recorded and then later transcribed by JV. Transcripts were not shared with participants and participants did not provide feedback on the findings.

### Analysis

Interviews were analyzed using MAXQDA software [29] and were coded using data-driven analysis techniques. We used a realist or essentialist interpretive framework, which reports on the experiences, meanings, and the reality of participants [30]. This approach allowed us to theorize motivations, experience, and meaning in a straightforward way, as we assumed a unidirectional relationship between meaning, experience, and language [30]. We assumed an ontological perspective wherein reality is seen as multiple through many views [31]. Our qualitative approach to inquiry was thematic analysis where we inductively identified emergent themes from the data [30]. Three researchers (JV, PW, and SRI) met several times to develop and refine the codebook. Two researchers (JV and PW) independently coded all the interview transcripts and then met to consensus code all transcripts. After coding was complete, the same three researchers met to collate the codes into potential themes and gather all data relevant to each potential theme through the synthesis of themes and convergent and divergent patterns. We then checked that the themes worked in relation to the coded extracts and the entire data set to generate a thematic map of the analysis. Finally, we refined the specifics of each theme, and the overall story of the analysis, generating clear definitions and names for each theme. We report out findings following the COREQ guidelines [32].

## Results

Out of the 64 patients approached, 52% declined participation due to limited time, or feelings of physical or emotional inability. A total of 31 participants were recruited from the Temmy Latner Centre for Palliative Care and their demographics appear in Table 1. Interview length was 15–30 min. Data were collected between July and September of 2018.

**Table 1:** Participant demographics

Characteristic	
*N*	31
Gender—*% (N)*	
Female	65 (20)
Male	35 (11)
Age—*M (SD)*	69 (12)^a^
Age range	33–99
Marital status—*% (N)*	
Single	19 (6)
Married	39 (12)
Divorced	29 (9)
Widowed	13 (4)
Highest level of education achieved—*% (N)*	
Some grade school	6 (2)
Some high school	3 (1)
High school graduate	13 (4)
Some postsecondary	3 (1)
Postsecondary certificate or diploma	20 (6)
Bachelor’s degree	26 (8)
Above bachelor’s degree	26 (8)
Other	3 (1)
Religious/spiritual affiliation—*% (N)*	
Atheism	6 (2)
Buddhism	6 (2)
Christian	42 (13)
Hinduism	3 (1)
Judaism	10 (3)
Spiritual	3 (1)
No religious or spiritual affiliation	17 (5)
Unsure	3 (1)

^a^One participant did not report their age

**Fig. 1:**
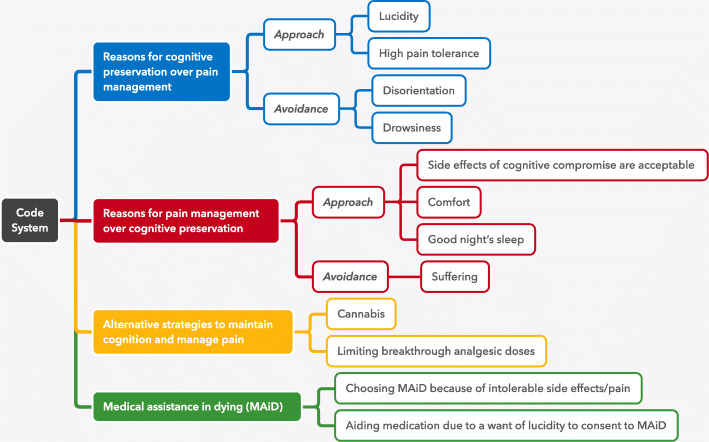
Thematic map of the analysis

Several themes emerged from this work (see Fig. 1): (1) Desire for cognitive preservation over pain control; (2) Desire for pain control over cognitive preservation; (3) Alternative strategies to pain management; and (4) The relationship of medical assistance in dying (MAiD) and pain management. For the first two themes, patients often framed their thoughts as either approaching the goal (e.g., “I choose cognitive preservation over pain management because I want to remain lucid”) or as avoiding the negative side effects (e.g., “I choose cognitive preservation over pain management because pain medication makes me intolerably drowsy”). We have structured our results for the first two themes to reflect these different framing techniques of “approach” and “avoidance.” While we present the results as a dichotomy between cognitive preservation and pain avoidance, in reality this is a much more nuanced distinction and a single patient may identify with both positions simultaneously—an approach-avoidance conflict.

### Cognitive preservation over pain management—approach

#### Lucidity

Of primary concern to many participants was retaining their lucidity and alertness. Many expressed a desire not to miss out on events due to the sedating effect of pain medication. For some, this was driven by wanting to be able to interact with loved ones or to remain able to participate in certain activities (e.g., driving).

*Patient: I want to be as awake as I can be. I want to be … well this is the next part of the journey, and I don’t want to miss out.*

*Interviewer: So your cognition was a factor when you were considering your treatment course?*

*P: Yes definitely. It’s the most important thing to me because I don’t have a lot of time but while I am here I want to be here [with my family].*

*— Interview 4*

*P: I want to have my wits about me, so that is a trade-off for me because I have things that I want to get accomplished and I don’t want to be rendered pain free but also be a zombie and not be able to articulate with friends and family and not be able to get things that I want to get done done.*

*— Interview 9*

#### High pain tolerance

Many participants stated they possessed a high tolerance to pain and therefore did not want or need medication to manage their pain. Some stated they avoided pain medications for many years, others that they would only take or consider taking medication if the pain was excruciating.

*First of all, I don’t take pain medication unless it’s absolutely necessary. I tolerate a fair bit of pain unless it’s excruciating and I must take something. So currently I am not taking any pain medication, but if you asked me a week ago, I was in excruciating pain and I was taking it.*

*— Interview 29*

### Cognitive preservation over pain management—avoidance

#### Balance/mobility

Concerns about balance and mobility were a commonly reported concern driving patients’ need for cognitive preservation. Many were unwilling to sacrifice their mobility in exchange for pain relief. Those that took pain medication and experienced disorientation reported the need for extra caution when getting around.

*P: Sometimes I worry about the effect [of the pain medication] on my mobility and if it puts me more at risk for a fall and losing my balance.*

*I: So do you feel like you are a bit out of balance?*

*P: Yes sometimes. It’s hard to say exactly what the cause is.*

*I: So how does this impact your daily functions?*

*P: I tread carefully.*

*— Interview 12*

*P: Well depending on the drug there are many side effects. Just as long as I can stand up without having the feeling of falling or anything like that and I am able to function.*

*I: So would something that impairs your cognitive abilities be okay for you to take?*

*P: To a degree yeah*

*I: Where would you draw the line?*

*P: It’s hard to say. As long as I am able to be mobile. I cant foresee any other side effects that really would affect me*

*— Interview 13*

#### Drowsiness

Drowsiness was a side effect many wished to avoid. Primarily, patients did not want to miss out on the time they had remaining, with some reporting that they would sleep for exceedingly long periods due to their pain medications. Some stated that they avoided certain activities (e.g., cooking and driving) after taking their pain medications, out of concern they and/or someone else may be hurt because of their drowsiness.

*Sleepiness and drowsy when I take it during the daytime. Mostly I take it in the morning and at night, I don’t take it during the day.*

*— Interview 7*

*P: Well sometimes it can make you a bit dim and sleepy, all of those things.*

*I: And do these negatively impact your day-to-day functioning?*

*P: Of course they do. I make decisions based on that. Like if I am drowsy then its not the best decision to drive a car, as I said. So I make decisions based on what medications I take and what the level of pain is.*

*— Interview 4*

### Pain management over cognitive preservation—approach

#### Side effects of cognitive compromise are acceptable

Several participants expressed that the side effects of pain medications were acceptable, given the relief from pain provided. Some explained they would be willing to tolerate side effects, if they were able to retain mobility and not be debilitated by pain.

*But no I think I am now at that point where I have to start saying ‘yes there is a trade off and yes it might make me a little foggy, and I will have to learn to live and compensate for that’. But I need to try and dampen down the pain.*

*— Interview 9*

*I would say so, because it’s no fun being in pain. I would talk to Dr. X – the dose I am at now seems to be working, but if at some point the dosage needs to be increased then I would let him know. If I have to increase the dosage and that comes along with some side effects, then I guess I will just have to live with the side effects. So long as the side effects are not so bad as to make me loopy all day and out of sorts. I would like to maintain my cognitive functions.*

*— Interview 21*

#### Good night’s sleep

The ability to sleep soundly at night was mentioned by a few participants as a desirable outcome of pain medications, as their pain levels would keep them up at night. One participant specifically mentioned their desire to sleep outweighed any concerns of side effects.

*It can make me dozy sometimes, which I like at nighttime especially to help me fall asleep.*

*— Interview 12*

#### Comfort

A few participants indicated they were willing to tolerate side effects to allow for certain levels of comfort.

*I: So you were thinking about your current quality of life and current pain management …*

*P: Yeah because the current quality of life … I was given a short about of life so you want it to be as high quality as possible.*

*— Interview 17*

*P: Well, through this thank god I have had minimal pain, except post-operatively. And I think what … if my pain were really severe and I needed enough analgesia to make me comfortable then definitely my husband and daughter would be designated by me to manage what they thought was best.*

*I: How do you think they would factor cognition and other side effects into that?*

*P: I think if it was all pre-arranged then they would go for my comfort.*

*I: So do you think they would say that you would be okay to sacrifice some cognitive abilities?*

*P: Yup. If I were to be in pain then yeah.*

*— Interview 16*

### Pain management over cognitive preservation—avoidance

#### Suffering

Avoidance of a feeling of suffering was a significant factor for taking pain mediation in many participants, with many being willing to sacrifice their cognition to avoid prolonged suffering.

*But last week I was screaming in pain and begging for a painkiller. I was really screaming. I didn’t care at that point if I had no cognition or whatever. If it were a constant exposure, then I am not sticking around. What is the use? We have assisted death in Canada, and it is not as if any of us in palliative care are getting any better.*

*— Interview 29*

*I think if the pain is not controlled by the doses you are using, and I were suffering from pain … but I think a lot of people that have severe pain or pain it just doesn’t go away … it’s not only the physical pain but it’s also the mental. It is very wearing and tiring.*

*— Interview 16*

*P: I cannot take the pain. I can’t sleep.*

*I: So you would rather have the [cognitive side] effects than the pain?*

*P: Yes.*

*— Interview 7*

### Alternative strategies

Patients described two alternative strategies to address the pain they were experiencing and find better balance between pain management and cognitive preservation. Some patients spoke about the use of cannabinoids to replace opioids for pain management, while others spoke of their efforts to limit breakthrough doses in order to preserve their cognition.

#### Cannabis

Several participants expressed an interest in the use of cannabis or cannabis derivatives to address pain. Interviews took place in July–September 2018—recreational cannabis was legalized in Canada in June 2018 [33] and available for purchase in October 2018, which may explain participants’ heightened interest.

*I am trying to get down on it. I would like to get to the point where I can flip it at least 50% over to cannabis. I have done cannabis before. But I would like to get to the point where I can use cannabis to substitute for morphine.*

*— Interview 25*

*I have been doing something there that I call an experiment, I have been using cannabinoid CBD oil. Is it working? I am a walking pharmacy, so quite frankly I don’t know whether any chemical agent is working in isolation or whether it is working synergistically with the others. But I know that the body is full of opioid receptors and a lot of people have said that there is evidence for [ … ] For example I have a sister-in-law in Dublin Ireland who uses CBD oil for her MS and swears by it. There is quiet a lot of information on the net about cannabinoids and MS but nothing really about ALS. I thought, you know what, nothing ventured nothing gained. And its organic, so I’m trying it.*

*— Interview 9*

#### Limiting breakthrough analgesic doses

Limiting breakthrough analgesic doses was reported by a few participants. These individuals would only take their breakthroughs in specific circumstances, as the side effects (e.g., cognitive impairment, constipation) were not often tolerable.

*I only ever take the medication when … well up until now before the pump I was taking a base dose of hydromorphone which would be in the background and then I had breakthrough immediate release hydromorphone for when its not cutting it and I need more. I was in control of … well the base dose I took regularly … but I was in control of the extra.*

*— Interview 4*

*But then again, I don’t make a habit of taking the breakthrough medication every day, only when I feel I need it. I take it and then I can go a whole week without taking it without needing additional intervention.*

*— Interview 21*

### Medical assistance in dying (MAiD)

Several participants expressed their feelings about the use of pain medication in the context of medical assistance in dying, which has been legal in Canada since June 2016 [34].

#### Choosing MAiD because of intolerable side effects/pain

Two participants expressed their desire to pursue medical assistance in dying if they reached a point at which their pain or other side effects were intolerable to live with.

*If the pain increases and becomes intolerable, I would have to take a look at my life at that point and decide if I want to stick around. I don’t want to become just something that is just sitting there in a daze. Having cognitive function is very important, that is all about quality of life.*

*— Interview 29*

*It also depends on how long. If it’s indefinite, then not very much. The kidney stone pain was extreme, and I wouldn’t want to tolerate that for more than half an hour. As soon as it looks like I have permanent, long term, significant pain, then I would go and apply for MAiD.*

*— Interview 28*

#### Avoiding medication due to a want of lucidity to consent to medical assistance in dying

Two other patients expressed a desire to pursue medical assistance in dying and were specifically avoiding pain medications that may lead to cognitive compromise, in order to satisfy the Canadian legal requirement that a patient be able to express consent immediately prior to their death via medical assistance in dying [34].

*[ … ] I am definitely wanting to pursue the idea of MAiD. In order to invoke that right now, we understand that the legislation is that one must be clear of mind at the moment of signing. And the trade off seems to be, from what I’ve read, that cancer patients say that they would forgo the pain medication in order to be clear of mind. It is a terrible trade off, and I hope over time that the particular clause gets reviewed and modified, because there is no reason for that.*

*— Interview 23*

*Yup. If I were to be in pain, then yeah. I know from the MAiD thing that unless you are cognitively okay then you can’t consent to the final blast.*

*— Interview 16*

## Discussion

Our interviews revealed four key themes: (1) Desire for cognitive preservation over pain control; (2) Desire for pain control over cognitive preservation; (3) Alternative strategies to pain management; and (4) the role of medical assistance in dying (MAiD) in pain management. The first two themes we structured according to approach and avoidance factors, highlighting the different ways in which patients approached the issue of pain management versus cognitive preservation. Understanding the balancing of approach and avoidance factors allowed for a more in depth understanding of how the patients in this study framed their decision-making processes.

Participants who favoured cognitive preservation over pain management indicated a desire to avoid the side effects of disorientation and drowsiness. They discussed how these side effects negatively impacted their quality of life. Importantly, in two other studies, the avoidance of unwanted adverse effects of opioids led many to report resisting opioid use until the pain was so severe that they felt they had no choice [13, 35]. It is unclear whether these participants’ avoidance lead them to a point where they had intolerable pain. We do know however, that patients who preferred pain management over cognitive preservation expressed that they favoured pain management to avoid undue suffering.

We heard from patients who preferred cognitive preservation that they highly valued maintaining lucidity and considered the side effects of disorientation and drowsiness intolerable. Patients have stated beliefs that taking morphine or other opioids may lead them to experience adverse effects more harmful to their quality of life and functioning than would occur if their pain was untreated [11].

Many participants favoured pain management over cognitive preservation and cited, as their rationale, that the side effects acceptable given the benefits, that they valued a good night’s sleep, and that they wanted comfort. These findings may relate to the palliative status of the patients, many of whom were at the end of life. Past research has alluded to the idea that many palliative care patients have a higher priority for relief from pain and symptoms than for cognition functioning [3], yet the subjectivity of this necessitates an emphasis on autonomy when balancing this decision.

An unexpected finding of our study was how pain management related to plans for the receipt of medical assistance in dying. Some participants felt medical assistance in dying was their last resort if the pain were to become too severe, while others avoided taking pain medications to maintain the lucidity necessary to consent to medical assistance in dying. Both of these themes have come up often in the clinical practice of our colleagues at the Temmy Latner Centre for Palliative Care; however, as far as we know, these themes have yet to be reported in the literature. Avoiding opioid analgesics and tolerating higher pain so as to maintain lucidity is an ethically complex medical concern.

An interesting omission from all our interviews was that none of the participants discussed the impact of their pain management or cognitive preservation on their informal caregivers. Both healthcare providers and patients have described a “good death” as one that which is pain-free, and where the symptoms are adequately controlled [35]. Consequently, poor pain control for patients towards the end of life has been shown to complicate the grief process for caregivers [35].

Decision making regarding pain management is complex, as demonstrated by the varied opinions expressed by participants. Some participants were willing to prioritize cognitive preservation, while others wanted to prioritize pain management. One factor which was curiously absent from most patient interviews we conducted was the role the healthcare provider plays in informing the patient’s decisions regarding pain management and cognitive preservation. Patients often structure their decisions by considering the opinions and suggestions of their healthcare providers [36]; however, few patients discussed having such conversations with their providers.

The potential for harm, misuse, and unwanted effects have led many clinicians to move away from using opioids as a first-line therapy for chronic non-malignant pain in efforts to avoid these impairments [10]. Indeed, in our study, patients discussed exploring cannabis as an alternative therapy, and the need to taper opioid breakthrough use to maintain lucidity. After cannabis was legalized via Bill C-45 in Canada in June 2018 [33], cannabis became a prominent topic in patients’ minds and was perceived by some patients as a viable alternative to opioids for pain treatment. Recent systematic reviews have shown the increasing interest of cannabis in medical use due to its multimodal action and the lack of negative effects that opioids carry [37]; however, a recent meta-synthesis concluded that for adults with advanced cancer, there was no difference between cannabinoids and placebo in pain scores and that cannabinoids had a higher risk of adverse events compared to placebo [38]. Importantly, there are alternatives to consider as part of the pain management approach in palliative care that address the non-nociceptive, psychosocial, and spiritual dimensions of pain in palliative care that are opioid insensitive with supportive psychotherapy as part of the provision of palliative care [37, 38].

### Broader context

Decision making regarding pain management in the palliative context can be compared to that of chronic pain management in non-palliative populations with some important contextual caveats. First, these decisions in palliative care are made in the compelling context of a life-limiting illness, in which individuals experience increasing pain with disease progression, but also face the reality of limited time to communicate with loved ones and maintain the cognitive capacity—even apart from opioid use—to attend to personal and financial affairs. Second, whereas guidelines carry a relatively strong recommendation for opioid use in the palliative care context [7], those for chronic pain management in the non-palliative population advocate use of non-opioid interventions as first choice and use of opioids only when their benefits and risks have been evaluated [10]. Third, in the non-palliative context, the pain management approach generally relies on setting realistic goals and achieving improvement in functional outcomes rather than pain intensity [39], whereas comfort may often take precedence over functionality in palliative care, particularly towards the last weeks of life. Nonetheless, patient concerns regarding opioid use and the degree of underuse adherence to prescribed opioid and other analgesic medications have been reported in non-palliative populations [40–44]. In a systematic review, non-adherence to prescribed pain medications, specifically in the form of their underuse, was reported with a weighted mean prevalence of 33% [42]. Underuse was positively associated with active coping strategies and the use of self medication [42]. Similar to patients in the cancer and palliative care contexts, studies of patients with chronic pain in non-palliative populations report non-adherent underuse of prescribed opioid [44] or opioid and other analgesic medications [40, 41, 43], which was associated with greater concerns regarding addiction [41] and side-effects of opioid and other analgesic medications [40, 41, 43].

The studies of pain medication adherence in non-palliative populations largely report medication side-effects collectively, making it difficult to determine the precise contribution of cognitive side-effects. A systematic review examining the cognitive effects of opioids on cognition in older adults with either cancer or non-cancer pain, reported mixed findings of both improvement and impairment of cognition [45]. An earlier systematic review of the cognitive effects of opioids in cancer reported an association between opioid use and poorer performance on neuropsychological testing, albeit of uncertain clinical significance [15]. Delirium is a distressing cognitive disturbance that occurs frequently in the palliative care population; a systematic review reported a delirium point prevalence of 35% on admission to an inpatient palliative care unit and a prevalence ranging up to 88% in the last hours to days before death [46]. One inpatient hospice study reported a prevalence rate of 9.8% for subsyndromal delirium, which may manifest as cognitive impairment without necessarily meeting the full criteria for a delirium diagnosis [47]. Although opioids have been identified as a frequent precipitant for delirium in the palliative care setting [48], the baseline vulnerability of the palliative care population due to advanced disease and the frequency of delirium as a naturally occurring pre-terminal event suggests that this population is at high risk of cognitive impairment regardless of opioid use. Both the subjective patient experience of milder cognitive difficulties experienced as part of a subsyndromal delirium and the memory of a previous episode of delirium with its frightening manifestations are likely to influence a patient’s decision making in terms of medication use.

### Limitations

Participants were recruited as a convenience sample screened by their physicians as eligible and “good” candidates for this study. These recommendations by physicians may have been biased. Unfortunately, we did not collect further clinical information on patients including clinical diagnosis, and whether their opioids were prescribed according to standard guidelines and if they used breakthrough pain medications. Given that the study was conducted in Toronto, a large and highly multicultural city, there are many patients in our program whose first language is not English. We were limited to recruiting English speaking participants, which may have prevented us from capturing an important aspect of this decision-making process. Further, we were limited to interviewing patients with capacity to consent, and some patients without capacity might have had limited capacity as a consequence of their opioid medications.

## Conclusion

The nature of palliative care is patient centered, so understanding the patients’ experiences and desires is crucial to improving palliative care services. Adopting a shared decision-making approach that incorporates an educational component on pain management, including medication side-effects [49], has the potential to promote patient empowerment and self-efficacy in pain management as part of high quality patient-centred care [50]. Findings from our study can help to inform patient decision-making regarding pain medications, and future studies should pilot interventions to better assist patients with this decision.

## Supplementary Information

**Additional file 1.** Appendix A—Semi-structured interview guide.

## Data Availability

Due to the potential for participant identification via the qualitative transcripts, and the lack of consent from participants for public sharing of their data outside of the research team, the data is not available upon request.

## Abbreviations

MAiD: Medical Assistance in Dying
