# Supplementary material for: “No thanks, I don’t want to see snakes again”: a qualitative study of pain management versus preservation of cognition in palliative care patients
Source: BMC Palliat Care. 2020 Nov 29;19:182. doi: 10.1186/s12904-020-00683-1 (PMC7702681; doi:10.1186/s12904-020-00683-1)
Supplement: Supplementary file 1 — Additional file 1. Appendix A—Semi-structured interview guide. [file 12904_2020_683_MOESM1_ESM.docx]

**Appendix**

## Appendix A—Semi-structured interview guide

Let me begin by reading something that is read to all participants. Pain is a common symptom experienced by a large number of palliative patients. While pain can be managed in a number of ways, a common side effect that patients experience is a decline in cognition functioning—such as feeling sleepy or “foggy”. How much pain someone is willing to tolerate versus what find of side effects they are willing to put up with varies with each person. Often patients and their healthcare providers must find the right balance between managing a patient’s pain and the cognitive side effects of those medications.

We are conducting a study to examine palliative care patient concerns regarding the trade-offs between optimal pain control and cognitive preservation. Our goal is understanding the factors that influence patient preferences for pain control at the cost of cognition, and what factors patients consider when making these decisions. These decisions are highly preference-sensitive, with no clear right or wrong answers—only the desires of the patient. The results of this study may then provide insight into how these decisions should best be communicated to patients to ensure that patient choices are in line with their personal preferences for pain control.

Please do not feel obligated to answer any questions you do not feel comfortable responding to, and please do not hesitate to ask any questions you may have at any point during the interview. Thank you for your participation. Do you have any questions before we begin, or would you like me to repeat anything?

1. How would you describe the level of pain you experience when taking your pain medication?
2. What effects does your pain medication provide? Is it primarily pain relief or are there other effects?
3. Do you currently experience any side effects of your pain medication?
   1. If yes, what are the side effects?
   2. Do these side effects negatively impact your day-to-day functioning?
      1. If yes, how do these side effects impact your life?
   3. Are these side effects tolerable given the pain relief they provide?
4. Do you feel that your pain is currently well controlled?
   1. If no, do you feel your pain medication should be increased?
   2. If you are currently experiencing cognitive side effects from your pain medication, are you willing to tolerate more/greater side effects in order to control your pain?
5. Overall, are you more willing to tolerate pain or cognitive side effects?
6. What factors did you consider when making decisions about your pain management medications?
   1. Did you consider the impact of pain medication on your cognition (i.e., your ability to reason and think clearly)?
      1. Why or why not?
